# Reoperation for isolated rheumatic tricuspid regurgitation

**DOI:** 10.1186/s13019-018-0793-7

**Published:** 2018-10-04

**Authors:** Younes Moutakiallah, Mahdi Aithoussa, Noureddine Atmani, Aniss Seghrouchni, Azeddine Moujahid, Abdedaïm Hatim, Iliyasse Asfalou, Zouhair Lakhal, Abdelatif Boulahya

**Affiliations:** 1 Cardiac surgery department, Mohammed V teaching military hospital, Hay Riyad, PB 10100, Rabat, Morocco; 2 Intensive care of cardiac surgery, Mohammed V teaching military hospital, Rabat, Morocco; 3 Cardiology department, Mohammed V teaching military hospital, Rabat, Morocco; 4 Faculty of medicine and pharmacy, Mohammed V university, Rabat, Morocco

**Keywords:** Isolated tricuspid regurgitation, Reoperation, Rheumatic

## Abstract

**Background:**

The reoperation for isolated tricuspid regurgitation in rheumatic population is rare and still unclear and controversial because of the rarity of publications. The aim of this study was to analyze short and long-term results and outcome of tricuspid valve surgery after left-sided valve surgery in rheumatic patients.

**Methods:**

Twenty six consecutive rheumatic patients who underwent isolated tricuspid valve surgery after left-sided valve surgery between January 2000 and January2017 were retrospectively registered in the study. The mean age was 48.2 ± 8.6 years with 8.3% as sex-ratio (M/F). EuroSCORE was 6.1 ± 5 (range 2.5 to 24.1). The mechanism of tricuspid regurgitation was functional and organic in respectively 14 (53.8%) and 12 cases (46.2%). Ten patients (38.5%) had previous tricuspid valve repair. Surgery consisted of 15 ring annuloplasty and 11 tricuspid valve replacement (5 bioprostheses and 6 mechanical prostheses). Follow-up was 96.1% complete, with a mean follow-up of 55.6 ± 38.8 months (range 1 to 165).

**Results:**

The operative mortality rate was 15.4% (*n* = 4) and the cumulative survival at 1, 5 and 10 years was respectively 80% ± 8%, 75.6% ± 8.7% and 67.2% ± 11.1% with no significant difference at 8 years between tricuspid valve replacement (80% ± 12.6%) and repair (57.6% ± 16.1%) (*p* = 0.5). Multivariable Cox regression analysis revealed that ascites (HR, 5.8; *p* = 0.01), and right ventricular dysfunction (HR, 0.94; *p* = 0.001) were predictors of major adverse cardiac events. There were no recurrence of tricuspid regurgitation and no structural or non-structural deterioration of valvular prostheses.

**Conclusion:**

The reoperation of rheumatic tricuspid regurgitation should be considered before the installation of complications such as right ventricular dysfunction and major signs of right heart failure. Despite the superiority of repair techniques, tricuspid valve replacement should not be banished.

## Background

Rheumatic heart disease (RHD) is still a serious problem of national health in our country with a significant cost and an enormous social and economic impact. This cost is widely expressed by redo valve surgery that usually requires several human and material resources, which is not easily available in underdeveloped areas like African countries. The reoperation for tricuspid regurgitation (TR) is the typical example of this redo valve heart surgery so much apprehended because of its operating risks related to the pathology itself, the surgical technique and the patient’s condition. In addition, this entity remains uncommon with many dark areas because of the rarity of publications in this field.

## Methods

After approval of our institutional review board, we retrospectively collected and analyzed the data of 26 consecutive patients who underwent isolated tricuspid valve surgery for neglected late TR appearing at distance of left-sided heart valve surgery (LSHVS) without tricuspid procedure or recurrent TR reappearing after tricuspid valve repair (TVrp) concomitantly performed with LSHVS. Data were extracted from preoperative and postoperative clinical notes, anaesthesia and operating data records, intensive care unit progress notes and laboratory data.

We used the term “redo” to describe the recurrent TR reappeared after tricuspid valve repair and the term “late” for the neglected TR appeared after LSHVS without tricuspid procedure. The term “functional” means TR secondary to the annular dilatation without involvement of the tricuspid leaflets as a result of increased pulmonary and right ventricular pressures consequently to mitro-aortic pathology. In opposition, the term “organic” describes the direct involvement of the tricuspid valve (TV) by the RHD [1, 2].

### Patients

The study included all patients (*n* = 26) operated in our institution for isolated “late” or “redo” rheumatic TR on a 17-year period between January 2000 and January 2017. Patients operated for non-rheumatic TR and patients operated for TR and any other concomitant valvular or bypass surgery were excluded from the study. Table 1 shows preoperative data. The average age at operation was 48.1 ± 8.6 years (range 29 to 63 years). Fifteen patients (57.7%) underwent tricuspid valve repair and 11 patients (42.3%) underwent tricuspid valve replacement (TVR) with 6 mechanical prostheses (32.1%) and 5 bioprostheses (19.2%). The New York Heart Association (NYHA) functional class was respectively 1, 2, 3 and 4 in 1 patients (3.8%), 4 patients (15.4%), 15 patients (57.7%) and 6 patients (23.1%). The mean interval from previous LSHVS and the current surgery was 164.1 ± 49.8 months (range 59 to 240 months) in the tricuspid valve repair group and 111.5 ± 72.1 months (range 6 to 241 months) in the replacement group (*p* = 0.04). Patients of repair group were significantly older than replacement group (51.2 ± 9.1 years vs 44 ± 0.9 years, *p* = 0.03); they had a longer delay between the two last surgeries (164.1 ± 49.8 months vs 111.5 ± 72.1 months, p = 0.04); they had also a larger tricuspid annulus (45 ± 3 mm vs 39.7 ± 7 mm, *p* = 0.02). In addition, there was significantly more likely to find a history of previous TV surgery in the replacement group (8 vs 2, *p* = 0.004), with a high proportion of organic TR with thickened leaflets and rheumatic lesions in the replacement group (10 vs 2, *p* < 0.001) and subsequently high percentage of tricuspid commissurotomy with or without Devega procedure (7 vs 1, *p* = 0.001).

**Table 1:** Preoperative characteristics of patients undergoing tricuspid valve surgery for isolated rheumatic tricuspid regurgitation (*n* = 26). Data are Presented as Mean ± SD, Median (Range), or *n* (%)

Characteristics	All patients *n* = 26	Functional TR *n* = 14	Organic TR *n* = 12	*p*-Value
Age (year)	48.2 ± 8.6	51.4 ± 9.3	44.4 ± 5.9	0.04
Sex (female)	24 (92.3%)	12 (85.7%)	12 (100%)	0.28
Symptoms duration (month)	29.5 ± 23.1	23.6 ± 16.5	36.3 ± 28.2	0.17
EuroSCORE	4.2 (2.5–24.1)	12.2 (2.5–24.1)	15 (3–16.2)	0.3
NYHA class 3–4	21 (80.8%)	11 (78.6%)	10 (83.3%)	0.58
Lower extremities edema	16 (61.5%)	9 (64.3%)	7 (58.3%)	0.54
Ascites	7 (26.9%)	4 (28.6%)	3 (25%)	0.60
Diabetes mellitus	3 (11.5%)	2 (14.3%)	1 (8.3%)	0.56
Gastro-duodenal ulcer	2 (7.7%)	2 (14.3%)	0 (0%)	0.28
History of stroke	3 (11.5%)	0 (0%)	3 (25%)	0.09
Haemoglobin < 12 g/dl	6 (23.1%)	3 (21.4%)	3 (25%)	0.60
Creatinine ≥2 mg/dl	2 (7.7%)	1 (7.1%)	1 (8.3%)	0.72
Atrial fibrillation	26 (100%)	14 (100%)	12 (100%)	1
Cardio-thoracic ratio	0.63 ± 0.09	0.62 ± 0.09	0.65 ± 0.09	0.4
Nature of tricuspid regurgitation				
- Late	16 (61.5%)	13 (92.9%)	3 (25%)	0.001
- Redo	10 (38.5%)	1 (7.1%)	9 (75%)	0.001
Tricuspid annulus diameter (mm)	42.8 ± 5.6	45.2 ± 3.1	40 ± 6.7	0.02
Tricuspid regurgitation severity	3.8 ± 0.4	3.7 ± 0.5	3.8 ± 0.4	0.5
- Grade 3	6 (23.1%)	4 (28.6%)	2 (16.7%)	0.65
- Grade 4	20 (76.9%)	10 (71.4%)	10 (83.3%)	0.40
Right ventricular dysfunction	12 (46.2%)	7 (50%)	5 (41.7%)	0.49
Systolic pulmonary arterial pressure (mmHg)	47 ± 16.4	41.7 ± 11.6	53.2 ± 19.3	0.09
Left ventricular ejection fraction (%)	57.1 ± 9.4	57.9 ± 6.7	56.1 ± 12.4	0.64
Left ventricular ejection fraction < 40%	1 (3.8%)	0 (0%)	1 (8.3%)	0.46
Left atrium diameter (mm)	55.9 ± 13.9	56.4 ± 17.1	55.4 ± 10.8	0.88
Number of previous heart operations	1.4 ± 0.6	1.2 ± 0.4	1.6 ± 0.8	0.2
- 1	18 (69.2%)	11 (78.6%)	7 (58.3%)	0.4
- 2	6 (23.1%)	3 (21.4%)	3 (25%)	1
- 3	2 (7.7%)	0 (0%)	2 (16.7%)	0.2
Previous left side valve surgery				
- Mitral valve replacement	16 (61.5%)	10 (71.4%)	6 (50%)	0.42
- Mitral and aortic valve replacement	10 (38.5%)	4 (28.6%)	6 (50%)	0.24
Previous tricuspid procedure				
- No tricuspid procedure	16 (61.5%)	13 (92.9%)	3 (25%)	0.001
- Devega technique	2 (7.7%)	1 (7.1%)	1 (8.3%)	1
- Devega + Commissurotomy	7 (26.9%)	0 (0%)	7 (58.3%)	0.001
- Commissurotomy	1 (3.8%)	0 (0%)	1 (8.3%)	0.5

*TR*: tricuspid regurgitation, *EuroSCORE*: European System for Cardiac Operative Risk Evaluation, *NYHA*: New York Heart Association

### Operative technique and data

All patients were electively operated under general anesthesia made by Cisatracurium besylate, Midazolam, Thiopental and Propofol. Twenty four patients (92.3%) were approached by median sternotomy and 2 patients (7.7%) by right anterolateral thoracotomy (4th intercostal space). Cardiopulmonary bypass was established in a conventional manner by central cannulation (ascending aorta and both vena cavae) and performed under moderate systemic hypothermia (32 °C). The tricuspid procedure was performed on arrested heart in 17 patients (65.4%) and on beating heart in 9 patients (34.6%) (*p* = 0.02), depending on surgical preference. Myocardial protection was achieved with antegrade cold (4 °C) crystalloid St. Thomas cardioplegia in 9 patients (34.6%) or antegrade cold (4 °C) blood high potassium cardioplegia in 8 patients (30.8%) (*p* = 0.05). The choice between tricuspid valve repair and replacement was made according to anatomical conditions with a preference for plasty techniques if suitable. Otherwise, we performed a tricuspid valve replacement by mechanical or biological prosthesis, which was inserted into the annulus with interrupted pledgeted mattress sutures using an everting suture technique. The native TV leaflets were left in place, preserving the subvalvular apparatus. In the septal area, the sutures were placed at the level of the leaflets avoiding the atrioventricular node injury. Conventional ultrafiltration was performed in 6 cases (23.1%). Fifteen patients (57.7%) underwent tricuspid valve repair, all by a Carpentier-Edwards (C-E) Semi-rigid Ring (Edwards Lifesciences, Irvine, CA), and 11 patients (42.3%) underwent tricuspid valve replacement. The mechanical prostheses used were 3 ATS Valve (ATS Medical Inc., Minneapolis, MN), 1 Sorin Bicarbon Slimline (Sorin Biomedica, Saluggia, Italy), 1 St. Jude Medical (St. Jude Medical, Inc) and 1 CarboMedics Valve (CarboMedics, Inc., Austin, TX). The biological prostheses used were 2 St. Jude Epic Biocor Valve (St. Jude Medical Inc), 2 Medtronic Hancock II Tissue Valve (Medtronic Inc., Minneapolis, MN) and 1 Sorin Pericarbon More (Sorin Biomedica, Saluggia, Italy). Table 2 summarizes operative data.

**Table 2:** Operative characteristics of patients undergoing tricuspid valve surgery for isolated rheumatic tricuspid regurgitation (n = 26). Data are Presented as Mean ± SD, Median (Range), or *n* (%)

Characteristics	All patients *n* = 26	Functional TR *n* = 14	Organic TR *n* = 12	*p*-Value
Median sternotomy	24 (92.3%)	14 (100%)	10 (83.3%)	0.20
Right thoracotomy	2 (7.7%)	0 (0%)	2 (16.7%)	0.11
Beating heart	9 (34.6%)	2 (14.3%)	7 (58.3%)	0.02
Cardioplegia	17 (65.4%)	12 (85.7%)	5 (41.7%)	0.02
- Cold crystalloid St Thomas cardioplegia	9 (52.9%)	7 (77.8%)	2 (22.2%)	0.62
- Cold blood high potassium cardioplegia	8 (47.1%)	5 (35.7%)	3 (25%)	0.62
Cardio-pulmonary bypass time (minute)	95.4 ± 39.7	87.9 ± 19.7	104.1 ± 54.4	0.35
Cross aortic clamping time (minute)	60 (35–170)	60 (38–170)	52 (35–103)	0.60
Hemofiltration (n, %)	6 (23.1%)	4 (28.6%)	2 (16.7%)	0.21
Mean ± SD (ml/Kg)	78.7 ± 34.6	89.7 ± 16.4	56.8 ± 16.2	0.58
Difficult weaning from cardio-pulmonary bypass	9 (34.6%)	3 (21.4%)	6 (50%)	0.22
Tricuspid valve repair:	15 (57.7%)	13 (92.9%)	2 (16.7%)	< 0.001
- Carpentier Edwards ring n°30	1 (3.8%)	1 (7.1%)	0 (0%)	0.9
- Carpentier Edwards ring n°32	11 (42.3%)	9 (64.3%)	2 (16.7%)	< 0.001
- Carpentier Edwards ring n°34	3 (11.5%)	3 (21.4%)	0 (0%)	0.03
Tricuspid valve replacement:	11 (42.3%)	1 (7.1%)	10 (83.3%)	< 0.001
- Mechanical prosthesis:	6 (23.1%)	0 (0%)	6 (50%)	< 0.001
- SJM n°27	1 (3.8%)	0 (0%)	1 (8.3)	0.87
- Sorin Bicarbon n°27	1 (3.8%)	0 (0%)	1 (8.3)	0.87
- ATS n°29	2 (7.7%)	0 (0%)	2 (16.7%)	0.7
- ATS n°31	1 (3.8%)	0 (0%)	1 (8.3)	0.87
- Carbomedics Valve n°31	1 (3.8%)	0 (0%)	1 (8.3)	0.87
- Biological prosthesis:	5 (19.2%)	1 (7.1%)	4 (33.3%)	< 0.001
- Sorin Pericarbon More n°27	1 (3.8%)	0 (0%)	1 (8.3%)	0.87
- SJM Epic Biocor Valve n°27	1 (3.8%)	0 (0%)	1 (8.3%)	0.87
- Medtronic Hancock Tissue Valve II n°29	2 (7.7%)	1 (7.1%)	1 (8.3%)	0.95
- SJM Epic Biocor Valve n°31	1 (3.8%)	0 (0%)	1 (8.3%)	0.87

*TR*: tricuspid regurgitation, *SJM*: St Jude Medical, *SD*: standard deviation

### Follow-up

Data was obtained from our local database. After discharge, all patients were included in our scheduled follow-up protocol with routine clinical controls at 1, 3, 6, and 12 months and annually afterwards. Follow-up data were provided either routinely by our outpatient clinic evaluation and telephone interviews with patients, relatives or referring physicians. The control was based on clinical examination, electrocardiogram, chest X-ray and echocardiography. The postoperative events and results were described according to the guidelines for reporting mortality and morbidity after cardiac valve interventions, approved by The Society of Thoracic Surgeons [3]. Follow-up was closed on September 30, 2017 and was 96.1% complete, with a cumulative duration of follow-up of 1746 patient-years and a mean follow-up period of 67.2 ± 46.7 months (range 1 to 165 months).

### Statistical analysis

The statistical analysis was performed using the IBM statistical package software for social sciences 19.0 (SPSS, Chicago, Illinois, USA). Data was presented as mean ± standard deviation (SD) or median (range) for continuous variables and n (%) for categorical variables. For the two group comparisons, chi-square test or Fisher’s exact test were used for categorical variables and either Student’s t-test or non-parametric Wilcoxon rank-sums test for continuous variables. Survival curves were constructed with the Kaplan-Meier method, and the Log-rank test was used for intergroup comparisons. Independent predictors of 30-day mortality and clinical outcomes were identified by Cox proportional hazard analysis. Predictors associated with a *p*-value of less than 0.2 on univariate analysis were considered in the multivariate analysis using stepwise selection. Results are expressed using hazard ratios (HRs). For all analyses, *p*-values < 0.05 were considered statistically significant.

## Results

### Immediate postoperative outcome

Postoperative events and results are described according to the guidelines for reporting morbidity and mortality after cardiac valve operations, approved by the Society of Thoracic Surgeons and The American Association for Thoracic Surgery. An early complication was defined as an event occurring after surgery during hospitalization, and a late complication as an event occurring after discharge. Reoperation is any operation that repairs, alters, or replaces a previously operated valve. A neurologic event includes any new, temporary, or permanent focal or global neurologic deficit. A bleeding event is any episode of major internal or external bleeding that causes death, hospitalization, or permanent injury or required transfusion. Cardiac complication was defined by the presence of one of the following: more than 72 h requiring an inotrope, return to operating room for bleeding or tamponade, new onset of atrial fibrillation, permanent pacemaker placement or in-hospital cardiac arrest. Respiratory complication was defined by the presence of one of the followings: duration of mechanical ventilation ≥24 h, re-intubation or tracheostomy. Infective complication was defined by the presence of one of the followings: pneumonia, sternal wound infection, mediastinitis or sepsis. Renal complication was defined by new onset renal failure, new onset renal replacement therapy [3].

The 30-day mortality was 15.4% with 4 early deaths. There was no significant difference between replacement group (*n* = 1; 9.1%) and repair group (*n* = 3; 20%) (*p* = 0.61). The causes of death were low cardiac output syndrome and multiorgan failure in 3 patients and a massive stroke in 1 patient. The early outcome and incidence of major postoperative complications are summarized in Table 3. There was no statistically difference between tricuspid valve repair and replacement concerning immediate outcome, with the same finding in the comparison between functional and organic TR.

**Table 3:** In-hospital outcomes of patients undergoing tricuspid valve surgery for isolated rheumatic tricuspid regurgitation (*n* = 26) with comparison between tricuspid valve repair group and tricuspid valve replacement group. Data are Presented as Mean ± SD, Median (Range), or *n* (%)

Characteristics	All patients *n* = 26	TV repair *n* = 15	TV replacement *n* = 11	*p*-Value
Ventilator support (hours)	12.5 (3–120)	9 (3–96)	17 (4–120)	0.47
ICU stay (hours)	71.4 ± 38.8	73.1 ± 44.4	69 ± 31.6	0.79
Postoperative stay (days)	16.9 ± 10.1	14.7 ± 10.8	19.8 ± 8.7	0.21
30-day mortality	4 (15.4%)	3 (20%)	1 (9.1%)	0.61
Low cardiac output syndrome	7 (26.9%)	4 (26.7%)	3 (27.3%)	1
Transitory renal failure	8 (30.8%)	5 (33.3%)	3 (27.3%)	0.54
Pneumonia	6 (23.1%)	4 (26.7%)	2 (18.2%)	1
Red Blood Cells transfusion > 1 unit	10 (55.6%)	5 (41.7%)	5 (83.3%)	0.15
Bleeding	6 (23.1%)	4 (26.7%)	2 (18.2%)	1
Reexploration for bleeding	3 (11.5%)	2 (13.3%)	1 (9.1%)	0.62
Sternal wound infection	1 (3.8%)	1 (6.7%)	0 (0%)	0.58
Sepsis	2 (7.7%)	1 (6.7%)	1 (9.1%)	1
Cardiac complication	11 (42.3%)	6 (40%)	5 (45.5%)	1
Respiratory complication	7 (26.9%)	4 (26.7%)	3 (27.3%)	0.66
Infective complication	7 (26.9%)	4 (26.7%)	3 (27.3%)	1
Renal complication	8 (30.8%)	5 (33.3%)	3 (27.3%)	1
Neurologic complications	1 (3.8%)	1 (6.7%)	0 (0%)	1

*TV*: tricuspid valve, *TVrp*: tricuspid valve repair, *TVR*: tricuspid valve replacement, *ICU*: intensive care unit

### Long-term outcomes

#### Late mortality

There were 3 late deaths (13.6%) at 6, 24 and 96 months. The causes of death were global cardiac failure and multiorgan failure. Two of the three deceased patients had a long history with RHD over more than three decades of disease progression with at least three cardiac surgeries; and all patients had a poor right ventricular function. The cumulative survival (calculated by Kaplan-Meier method) at 1, 5 and 10 years was respectively 80 ± 8%, 75.6 ± 8.7% and 67.2 ± 11.1%, with no significant difference at 10 years between tricuspid valve replacement (80% ± 12.6%) and tricuspid valve repair (60% ± 14.8%) (*p* = 0.52). Similarly, there was no significant difference between functional TR (77.1% ± 11.7%) and organic TR (48.5% ± 21.7%) (*p* = 0.41), and, on the other hand, between redo TR (55.6% ± 16.6%) and late TR (75 ± 13.6%) (*p* = 0.16).

**Fig. 1:**
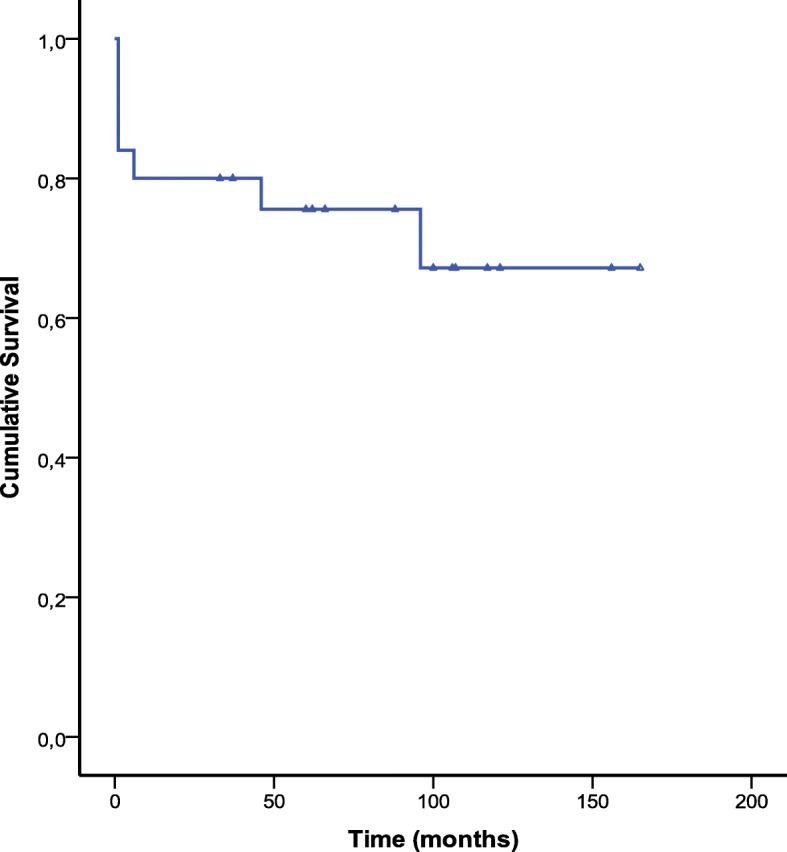
Kaplan-Meier curve of survival in patients who underwent reoperation for isolated rheumatic TR

**Fig. 2:**
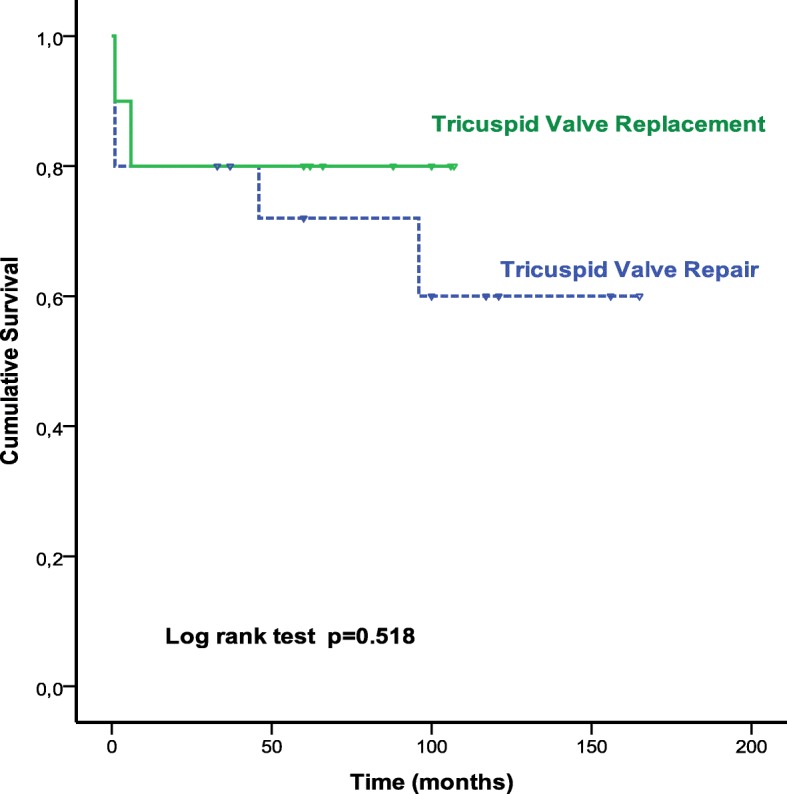
Kaplan-Meier curves comparing survival in patients who underwent tricuspid valve replacement versus tricuspid valve repair

**Fig. 3:**
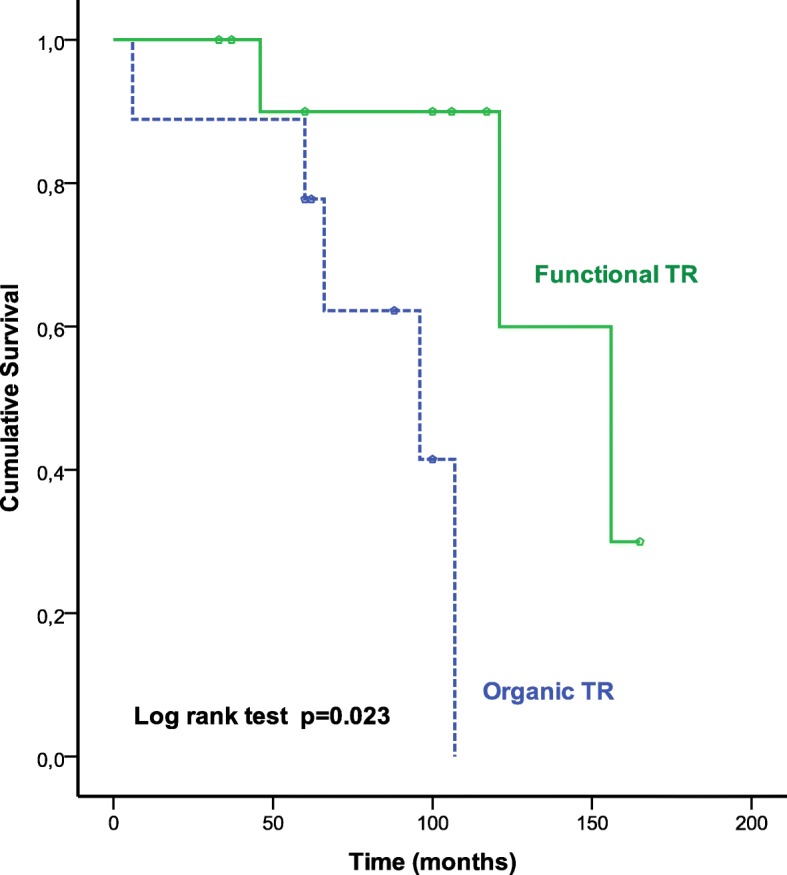
Kaplan-Meier curves comparing survival in patients with Functional TR versus Organic TR

Univariate analysis identified the nature organic of the TR, NYHA class, EuroSCORE > 8, anemia, ascites, systolic pulmonary artery pressure > 60 mmHg, right ventricular dysfunction, postoperative bleeding, blood transfusion and cardiac complications as significant predictors of overall mortality. On multivariable Cox regression analysis, ascites (HR, 5.8; *p* = 0.01) and right ventricular dysfunction (HR, 0.94; *p* = 0.001) were independent predictors of overall mortality (Figs. 1, 2, 3 and Table 4).

**Table 4:** Independent predictors of overall mortality after reoperation for isolated rheumatic tricuspid regurgitation

Characteristics	Univariate Analysis			Multivariate Analysis		
	HR	95% CI	*p*-Value	HR	95% CI	*p*-Value
EuroSCORE > 8	0.03	0.001–0.68	0.03^a^	0.6	0.33-1.11	0.1
NYHA class	0.25	0.05–1.24	0.09^a^	0.28	0.01-9.16	0.48
Anemia	0.27	0.04–1.86	0.18^a^			
Organic TR	7	0.5–98.6	0.15^a^			
Ascites	0.06	0.004–1.04	0.05^a^	5.8	1.25-9.26	0.01^b^
SPAP > 60 mmHg	0.95	0.88–1.03	0.19^a^			
RV dysfunction	0.2	0.03–1.35	0.09^a^	0.94	0.004-1.43	0.001^b^
Postoperative bleeding	0.21	0.03–1.95	0.18^a^			
Blood transfusion	0.14	0.013–1.63	0.12^a^			
Cardiac complications	0.14	0.01–2.01	0.15^a^			

*HR*: hazard ratio, *CI*: confidence interval, *TR*: tricuspid regurgitation, *SPAP*: systolic pulmonary arterial pressure, *RV*: right ventricle

^a^: *p*-Value < 0.2 (for univariate analysis);

^b^: p-Value < 0.05 (for multivariate analysis)

#### Late reoperation

Of the 19 patients who survived the surgery, no patient needed a reoperation for TV disease or any other cardiac condition. There were no recurrence of TR and no structural or non-structural deterioration of valvular prosthesis. The 10-year event-free survival rate was 36.5% ± 18.2% and was significantly higher in repair group (50% ± 23%) in comparison with replacement group (0%) (*p* = 0.04).

All patients had mechanical mitral valve prostheses and, consequently, received oral anticoagulation by acenocoumarol without systematic use of platelet aggregation inhibitors. The target International Normalized Ratio (INR) ranged between 3 and 4. We recorded no thromboembolic events and 2 patients had hemorrhagic episodes related to over-anticoagulation (recurrent epistaxis and spontaneously resolving psoas hematoma).

Of the 18 controlled survivors, 16 patients (88.9%) were in NYHA class 1–2 and 2 patients (11.1%) were in class 3. All patients expressed an improvement of their functional status. Two patients (11.8%) had signs of right heart failure requiring enhanced medical treatment. All patients maintained atrial fibrillation as cardiac rhythm, and no patient experienced conductive disorder or needed a permanent pacemaker insertion in early or long-term period. The mean cardio-thoracic ratio on the chest x-ray was 0.63 ± 0.1 (range 0.59 to 0.7).

The assessment of TR was done only by echocardiography and no patients had cardiac catheterization. According to the most recent echocardiography, no controlled patient had moderate or severe TR. There was no case of structural or non-structural dysfunction of implanted tricuspid valvular prostheses or rings. However, 1 patient had elevated mean gradient of aortic valvular prosthesis above 35 mmHg.

## Discussion

The TR appearing after LSHVS should be considered differently depending on whether it is a repeat TV operation (redo TR) or not (late TR). In our experience, late TR is dominated by functional mechanism with normal leaflets and dilated tricuspid annulus; whereas, redo TR is dominated by the organic rheumatic origin with direct involvement of the TV components by abnormal thickening of the leaflets, adhesion of the commissures and shortness of chordae. This concept influenced significantly the type of tricuspid valve surgery with significant dominance of tricuspid valve repair for “late” and “functional” TR, and high proportion of replacement for “redo” and “organic” TR.

The management of TR in rheumatic patients remains controversial with many shadows. If the severe TR rises no doubt about the need of surgical correction concomitantly with LSHVS, the decision for moderate and mild TR remains uncertain and not unanimous. Some authors suggest no treatment, hoping the return of pulmonary pressures to acceptable levels making the TR “spontaneously” disappear, or at least stabilize at a non-significant level; believing the dogma that “functional” TR will subside after appropriate LSHVS [4]. However, the annular dilatation is a progressive process and may not be accompanied by TR initially, but eventually leads to it [4]. In our study, all patients with functional TR had at the time of the initial operation at most a mild TR with normal or non significant dilated annulus at the preoperative echocardiography; and the TR appeared or worsted progressively with significant annular dilatation. Antunes suggested as risk factors for persisting or worsening TR after a mitral valve procedure without TV surgery, the persistence or recurrence of mitral valve disease, longstanding right ventricular dilatation [4]. Xiao mentioned five main factors of the Late TR progression after LHVS: the persistence of pulmonary hypertension, the right ventricle tricuspid valvular disproportion, the atrial fibrillation, the progression or development of rheumatic lesions and DeVega’s suture annuloplasty technique [5]. In our experience, we found other risk factors: female sex, major left atrium dilatation, pulmonary hypertension and organic TR.

For patients with previous tricuspid valve repair (redo TR), the failure of the primary plasty were mainly due to direct rheumatic involvement of the TV where the progressive worsening of the TR is more evident and faster. Additionally, in this case, TR was usually associated with some degree of stenosis where the leaflets were thick, immobile and rigid; the commissures were fused, the chordea were short and agglutinated and the annulus was deformed. On 10 patients of Redo TR, 90% had organic rheumatic TV disease with 8 cases of combined Devega procedure and tricuspid commissurotomy, 1 case of Devega procedure alone, 1 case of tricuspid commissurotomy alone and no case of ring annuloplasty. Then, we think a posteriori that Devega procedure was wrongly used in this group of severe patients with organic TR where an aggressive attitude with rigid ring annuloplasty to remodel the deformed tricuspid annulus was probably more appropriate and accurate. Actually, we changed our policy towards organic TR and we performed since 2005 systematically a rigid ring annuloplasty with tricuspid commissurotomy if needed. Devega procedure is reserved to moderate functional TR with mildly dilated annulus.

It is true that since its first description in 1969, the tricuspid valve replacement has acquired the reputation of bad operation with increased incidence of thromboembolic event for the mechanical prostheses and degeneration problems of bioprostheses. However, it must be said that the prostheses used in this setting were of the older generations with many problems even in mitro-aortic position.

Generally, in valve heart surgery, valve repair techniques had shown their superiority to valve replacements. This concept is more patent in TV surgery for several reasons: first of all, TV surgery is usually done in multivalvular patients with mitro-aortic prosthesis; secondly, prosthesis in tricuspid position had shown their limits in many studies with high risk of complications and, finally, the TV tolerates well a less than perfect repair contrasting with mitro-aortic position [4].

However, repairing an organic rheumatic TV disease with abnormal leaflet, commissures and chordae, is not always possible. In some cases, we insist to repair a deeply pathological valve at the expense of a significant risk of plasty failure and recurrence of TR while a valve replacement could solve the problem effectively with relatively acceptable risk of related-valves complications especially with the new generation of prosthesis. We should define objectively good candidates either for tricuspid valve repair or replacement in rheumatic population. In addition, repair techniques of deeply affected rheumatic valves require much more dexterity and surgical experience, which is not easily available for all, especially young surgeons and low volume centers.

For tricuspid valve replacement, bioprostheses were initially considered ideal because they would not require anticoagulation and were expected to have a slower degeneration than in the mitral or aortic position [6]. However, a Nakano review of the Carpentier-Edwards pericardial bioprosthesis reported non-structural dysfunction in 72.8% of patients by pannus formation on the ventricular side of the cusps. Control echocardiography revealed an incidence of pannus in 35% of patients with at least 5 years of follow-up [7]. Guerra reported similar changes on explanted porcine Hancock valves with the presence of a pannus on the ventricular side of the cusps limiting their flexibility and function [8]. The same finding was supported by Carrier’s work [9]. Rizzoli in his meta-analysis suggested that mechanical prostheses should be preferred in young patients and in patients with left sided mechanical prostheses [6]. A meta-analyse of Kunadian, involving 561 articles and more than 1000 mechanical and biological tricuspid prostheses, confirmed that there is no significant difference in survival and the re-operation for bioprosthetic degeneration remains equivalent to the re-operation for thrombosis of mechanical prostheses. In addition, the study showed that 95% of patients with bioprostheses continue to receive anticoagulation [10].

In rheumatic condition, we believe that new generation of mechanical prostheses are at least non inferior to bioprostheses in tricuspid position, because patients need effective oral anticoagulation for mechanical prostheses on the mitro-aortic position with enlarged cardiac chambers and atrial fibrillation. So, the main indication and the principal benefit of bioprostheses is absent with additional risk of structural deterioration and re-operation, which is not negligible for multi-operated patients and in low income population like in African countries. Recently, we have changed our therapeutic strategy regarding the choice of TV prosthesis; when it is about isolated TR without involvement of mitro-aortic valves (like traumatic, infectious and congenital TR …etc), we follow the usual guidelines concerning heart valves replacement policy. However; in case of rheumatic polyvalvulopathy, we prefer now new generation of mechanical prosthesis for several raisons: our patients are mostly young, with mitral or mitro-aortic mechanical prostheses, dilated left atrium and atrial fibrillation.

Reoperation for TR is associated with high operative risk because of its high operative risk related to the pathology itself, the condition of the patient and the risk of thoracic re-entry [11]. The results of this surgery were poor with high rates of mortality, which might reach 10–20% [4]. However, the mortality had dropped to acceptable levels in the recent reports [12–14] thanks to preoperative preparation, medical therapy, physiotherapy, myocardial protection, anesthetic and surgical techniques [4]. Major signs of right heart failure, such as ascites and right ventricular dysfunction, were significant predictors of morbid-mortality in our study. We can add other risk factors like high functional class and pulmonary hypertension.

### Limitations

This study is mainly limited by its retrospective design and relatively small cohort size. In addition, it is limited by the heterogeneity of mechanical and biological prostheses. Consequently, we cannot draw strong conclusions about this subject especially in absence of control group. However, in many ways our study is original, because, at the expense of a limited number of patients, we focused on the reoperation for isolated rheumatic TR which constitutes a homogeneous group, with particularities that differentiate it from other etiologies of TR and the relative rarity of articles that treat the subject.

## Conclusion

The reoperation of rheumatic TR should be considered before the installation of complications such right ventricular dysfunction and major signs of right heart failure. Despite the superiority of repair techniques, tricuspid valve replacement should not be banished.

## Abbreviations

LSHVS: Left-sided heart valve surgery
RHD: Rheumatic heart disease
TR: Tricuspid regurgitation
TV: Tricuspid valve
TVR: Tricuspid valve replacement
TVrp: Tricuspid valve repair
